# Exploring the Holiday Effect on Air Temperatures

**DOI:** 10.1038/s41598-018-36351-x

**Published:** 2018-12-18

**Authors:** Shaojing Jiang, Kaicun Wang

**Affiliations:** 1 State Key Laboratory of Earth Surface Processes and Resource Ecology, College of Global Change and Earth System Science, Beijing Normal University, Beijing, 100875 China; 2 School of Forestry and Environmental Studies, Yale University, New Haven, Connecticut 06511 USA

## Abstract

Anthropogenic emissions are generally lower during holidays than they are on workdays, this pattern is expected to result in temperature variations. Variations in the daily maximum (*T*_*max*_), mean (*T*_*mean*_) and minimum (*T*_*min*_) air temperatures and the diurnal temperature range (DTR) during the Chinese New Year holiday are evaluated with two methods using daily meteorological observations collected at 2200 stations in China from 1961 to 2015. These two methods yield nearly equivalent results that reflect strong variations in the defined holiday effects. During the period from 1961 to 1980, *T*_*mean*_, *T*_*max*_, *T*_*min*_ and the DTR all exhibit cooling holiday effects, this effect as measured by the DTR disappears during the period from 1981 to 2000. However, during the period from 2001 to 2015 warming holiday effects are observed for *T*_*max*_ and the DTR. The evaluation shows that the holiday effect is neither unique nor statistically significant. These results indicate that the holiday effect is primarily caused by natural atmospheric oscillations, because ΔT oscillates noticeably with periods of approximately 7.1 days, 8.5 days and 16.2 days, and these oscillations can account for approximately 75.6% of the variance in ΔT. The oscillation identified here is consistent with the fundamental theory of Rossby wave in the atmosphere.

## Introduction

Whether regional and global climate changes can be attributed to natural and anthropogenic causes is a central issue^1,2^. The effects of holidays and weekends on meteorological and environmental variables (i.e., the differences in aerosol concentrations and air temperatures across days that fall on workdays vs. across days that fall on weekends or holidays) represent an important aspect of the detection and attribution of climate change. These effects have been analyzed in several studies using observed meteorological and satellite datasets^3–5^.

The effects of holidays and weekends on meteorological and environmental variables have been attributed to anthropogenic causes for decades. Human activities, such as commercial transportation and industrial activities, occur with reduced intensities on weekends relative to weekdays in most countries^6^. Thus, researchers have generally focused on anthropogenic impacts while assuming that the weekly cycles of consumption and emissions resulting from human activity could help explain the effects of weekends on the climate^7^.

The first investigation of the potential relationships between human activities and weekends was conducted by Ashworth *et al*.^8^, who argued that weekly cycles of smoke and hot gas emissions from factory chimneys led to reductions in rainfall on Sundays. Choi *et al*.^9^ found an relationship between weekly anthropogenic air pollutant emissions and meteorological conditions over China at a regional scale and suggested a possible link between the formation of clouds and the weekly variations in the mass concentrations of atmospheric aerosols^10^. On a larger scale, Gordon^11^ analyzed the weekly cycles of meteorological variables in the Northern Hemisphere and observed that the average daily temperature was lowest on Sunday and highest on Wednesday from the period of 1979 to 1992. Ohashi *et al*.^12^ calculated the weekend effect on the air temperatures over the city of Osaka in Japan from 2013-2014 and found that the daytime temperatures were more than 1 °C higher on weekdays than on weekends because of the anthropogenic electricity use.

In addition to weekly cycles, other short-term oscillations of atmospheric phenomena with different periods have been widely reported. Espy and Witt^13^ studied mesospheric temperatures in Scandinavia from June to August 1992 and identified a significant quasi-16-day temperature oscillation pattern. Jiang *et al*.^14^ found that zonal mean temperatures oscillate with periods of approximately 9 and 13.5 days. Such oscillations have been attributed to multiple natural processes, such as the North Atlantic Oscillation (NAO) and Rossby waves. The NAO is a large-scale meridional oscillation of atmospheric mass that occurs between the subtropical anticyclone near the Azores and the subpolar low pressure system, which is related to a wide range of climate variation over the Northern Hemisphere across a variety of time scales^15,16^. Rossby waves are long-period turbulent waves that propagate both within the atmosphere and over the oceans, and display considerable variations in their length and time scales^17–19^.

The impacts of natural factors (e.g., the NAO and Rossby waves) on multiple meteorological elements, including temperature and precipitation, have been extensively investigated^20,21^. The NAO has been regarded as a dominant pattern that can significantly impact both temperature and precipitation with a time scale of greater than 10 days^22,23^. Hurrell^24^ concluded that the temperature variabilities over Scandinavia and central Asia are strongly related to NAO. Delworth and Zeng^25^ argued that NAO fluctuations can modulate hemispheric temperature by several tenths of a degree.

Studies have also examined the relationship between Rossby waves and meteorological elements^26^. Morioka *et al*.^27^ found that the temperature variabilities in the South Atlantic and Indian Oceans may be related to quasi-stationary oceanic Rossby waves. Kikuchi and Wang^28^ argued that Rossby wave trains play an essential role in the initiation, development, and propagation of quasi-biweekly oscillation in air temperature over the globe.

However, whether natural or anthropogenic factors cause the weekend effect is still debated^6^. Kim *et al*.^29^ attempted to compare the influences of natural and anthropogenic factors on the weekend effect using diurnal temperature data from 1950 to 2000 and concluded that the natural weekend effect induced by Rossby waves was much stronger than the anthropogenic weekend effect. Godfrey *et al*.^30^ suggested that meteorological patterns occurring frequently on or near a specific calendar date are more likely to be statistical phantoms than genuine climatic phenomena.

**Figure 1:**
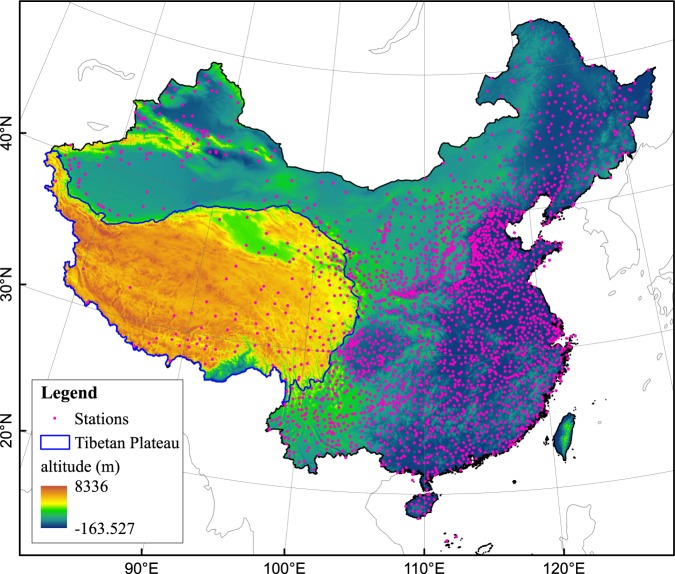
The locations of the 2200 meteorological stations in China and the topography of the study area.

The reductions in anthropogenic emissions that occur during major holidays, such as the Chinese New Year holiday, provide good opportunities to study the impacts of holidays on observed air temperatures. The Chinese New Year holiday is a national week-long holiday that has been celebrated for centuries. It provides a good opportunity to investigate the holiday effect on observed air temperatures. Based on daily air temperature observations at 2 meters above the surface collected at 2200 weather stations in China from 1961 to 2015 (see Fig. 1), this study evaluates the effects of the Chinese New Year holiday on air temperatures.

## Results

### Variations in the Holiday Effect

**Figure 2:**
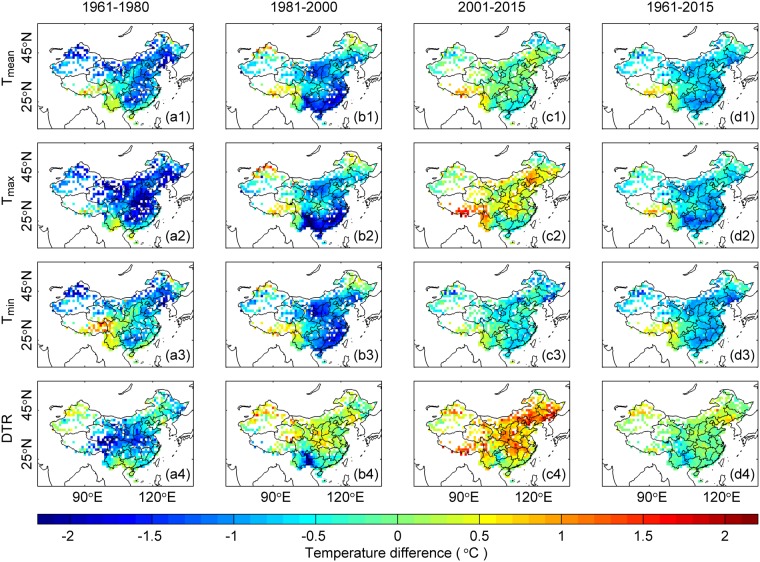
Spatial distribution of the holiday effect derived from *T*_*max*_, *T*_*mean*_, *T*_*min*_ and the DTR in China using Method 1 during 1961–1980 (a1, a2, a3, and a4), 1981–2000 (b1, b2, b3, and b4), 2001–2015 (c1, c2, c3, and c4) and 1961–2015 (d1, d2, d3, and d4). Using Method 1, the difference between the air temperatures during the holiday week and during the work week immediately preceding and following the holiday is regarded as the holiday effect. The data on all of the available days are used.

Figure 2 displays the spatial distribution of the holiday effect as determined using Method 1 (see Methods Section for details), which directly compares the differences in air temperatures during the week of the Chinese New Year holiday with those during the work week immediately preceding and following the holiday week. The corresponding holiday effect values averaged over all of the stations are shown in Table 1.

**Table 1:** The simple spatial average and area-weighted average values of the temperature differences derived from *T*_*mean*_, *T*_*max*_, *T*_*min*_ and the DTR during the different periods in China.

Temperature	Method	Average	All available days				Dry days			
			1961–1980	1981–2000	2001–2015	1961–2015	1961–1980	1981–2000	2001–2015	1961–2015
*T*_*mean*_	Method 1	Simple spatial	−0.83	−0.91	−0.10	−0.65	−0.45	−1.10	−0.13	−0.57
		Area-weighted	−0.76	−0.73	−0.06	−0.56	−0.48	−0.86	−0.09	−0.48
	Method 2	Simple spatial	−0.72	−0.81	0.06	−0.52	−0.41	−0.98	0.11	−0.44
		Area-weighted	−0.67	−0.64	0.06	−0.46	−0.45	−0.74	0.10	−0.37
*T*_*max*_	Method 1	Simple spatial	−1.22	−0.92	0.28	−0.67	−0.49	−1.26	0.22	−0.56
		Area-weighted	−1.12	−0.72	0.31	−0.57	−0.59	−0.96	0.27	−0.46
	Method 2	Simple spatial	−1.05	−0.70	0.26	−0.54	−0.45	−1.03	0.40	−0.40
		Area-weighted	−0.95	−0.52	0.33	−0.43	−0.52	−0.75	0.45	−0.30
*T*_*min*_	Method 1	Simple spatial	−0.53	−0.92	−0.40	−0.64	−0.45	−0.98	−0.40	−0.58
		Area-weighted	−0.49	−0.74	−0.37	−0.55	−0.43	−0.77	−0.37	−0.50
	Method 2	Simple spatial	−0.49	−0.94	−0.13	−0.55	−0.43	−0.98	−0.13	−0.50
		Area-weighted	−0.49	−0.76	−0.19	−0.51	−0.43	−0.77	−0.19	−0.46
*DTR*	Method 1	Simple spatial	−0.69	0.01	0.67	−0.03	−0.04	−0.28	0.62	0.03
		Area-weighted	−0.64	0.02	0.68	−0.02	−0.16	−0.19	0.64	0.04
	Method 2	Simple spatial	−0.56	0.24	0.39	0.01	−0.02	−0.05	0.54	0.10
		Area-weighted	−0.46	0.24	0.52	0.08	−0.09	0.03	0.64	0.16

Method 1 directly compares the air temperatures between the week of the Chinese New Year holiday and the work week immediately preceding and following the holiday week. Method 2 evaluates the average daily deviation in air temperatures (ΔT) during the holiday week. The results obtained using data on all of the available days and on dry days only are compared (units: °C).

During the period from 1961 to 2015, the holiday week exhibits cooling with respect to *T*_*mean*_, *T*_*max*_ and *T*_*min*_ (see Fig. 2d1,d2 and d3), the magnitudes of which are −0.65 °C, −0.67 °C, and −0.64 °C (see Table 1), respectively. Among all of the stations, the holiday effect values for *T*_*mean*_, *T*_*max*_ and *T*_*min*_ range from −3.42 °C to 3.35 °C. With the exception of the Tibetan Plateau (the blue circle area in Fig. 1), most of the regions in China exhibit cooling holiday effect. However, the holiday effect of DTR is almost zero during 1961–2015 (see Fig. 2d4).

To reduce the impact of a heterogeneous station distribution, both area-weighted and simple spatial average values over China are calculated. The simple spatial average reflects the average of the holiday effect among all of the stations. Meanwhile, the area-weighted average is calculated while considering area weights; in this approach, a single 1° × 1° grid cell can contain several stations or none. The holiday effect in each grid cell is the average of the stations in that grid cell. The area-weighted average is the sum of the holiday effect in each grid cell multiplied by its area weight (the area weight of one grid cell varies with the latitude), which is the area of the grid cell multiplied by the total area of all of the grid cells.

Both simple-spatial averages and the area-weighted averages of the holiday effect are shown in Table 1. The area-weighted averages are approximately 0.1 °C weaker than the simple spatial average values. Considering the rapid increase in anthropogenic activity following the 1978 economic reform program in China^31^, the holiday effect values obtained for three separate sub-periods, namely, 1961–1980 (see Fig. 2a1,a2 and a3), 1981–2000 (see Fig. 2b1,b2 and b3) and 2001–2015 (see Fig. 2c1,c2 and c3), are also compared to investigate the anthropogenic impacts on the observed air temperatures. The whole procedure in the Methods Section is done for each sub-period to analyze the holiday effects and their statistical significances.

During the period from 1961 to 1980, *T*_*mean*_, *T*_*max*_, *T*_*min*_ and the DTR all demonstrate cooling holiday effects (see Fig. 2a1,a2,a3 and a4), which are −0.83 °C, −1.22 °C, −0.53 °C and −0.69 °C (see Table 1), respectively. Spatially, most of the regions in China exhibit cooling holiday effects with the exception of the southeastern region.

During the period from 1981 to 2000, the cooling holiday effect becomes stronger in terms of *T*_*mean*_ and *T*_*min*_, which are −0.91 °C and −0.92 °C, respectively, whereas the cooling holiday effect in terms of *T*_*max*_ (−0.92 °C) becomes weaker. Additionally, the cooling holiday effect nearly disappears when the DTR (0.01 °C) is considered. A warming holiday effect is observed over the Tibetan Plateau (i.e., the southwestern region of China) and along the northern border of Xinjiang Province (i.e., the northwestern region of China).

During the period from 2001 to 2015, the cooling holiday effect is once again weaker with regard to *T*_*mean*_ and *T*_*min*_ (−0.1 °C and −0.4 °C, respectively). More importantly, the holiday effects of *T*_*max*_ (0.28 °C and 0.67 °C, respectively) and the DTR become opposite from each other. Similarly, warming holiday effects are observed with regards to *T*_*mean*_, *T*_*max*_, *T*_*min*_ and the DTR over the Tibetan Plateau; however, in Xinjiang Province, the warming holiday effect is only obvious in the DTR.

**Figure 3:**
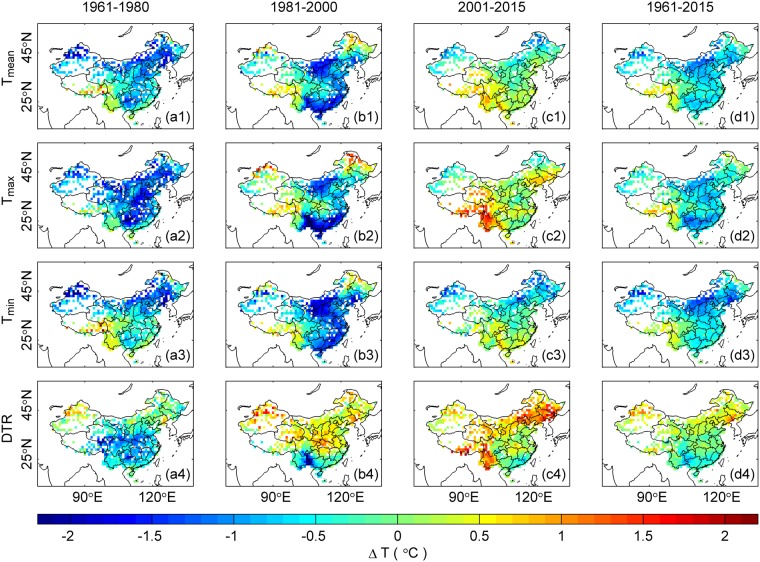
Same as Fig. 2, except that Method 2 is used. In this method, the holiday effect is the average of ΔT (i.e., the temperature difference between the original daily and fitted temperatures, refers to Fig. 7b) during the holiday week. The coherent spatial patterns in the holiday effect throughout China are similar to those observed in the results obtained from Method 1 (Fig. 2).

Figure 3 shows the same data as Fig. 2, except that the holiday effect is evaluated using Method 2 (see Methods Section for details). The spatial patterns of the holiday effect (see Fig. 3) with respect to *T*_*mean*_, *T*_*max*_, *T*_*min*_ and the DTR are similar to those using Method 1 (see Fig. 2). These two methods produce nearly equivalent results (see Table 1).

### Confidence Tests

Method 1 allows us to investigate the inter-annual variabilities in the holiday effect. A two-tailed *t*-test is employed to test the significance of the holiday effect. The FDR approach at *p* = 0.01 or *p* = 0.05 level is applied to the *p*-values to obtain the significant results at different confidence levels. Almost no stations (<0.04%) are significant whether at *p* = 0.01 or *p* = 0.05 level [see supplementary Table S1].

**Figure 4:**
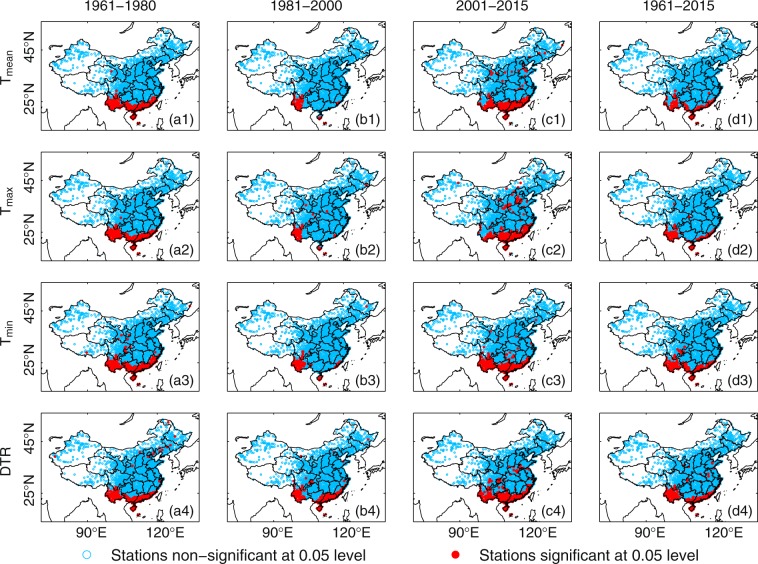
Map of stations that are significant (red dots) or non-significant (blue dots) at the *p* = 0.05 confidence level compared with those on other days throughout the year according to the Monte Carlo significance test. The significance of the holiday effect in *T*_*mean*_, *T*_*max*_, *T*_*min*_ and the DTR is investigated. Several different periods, i.e., 1961–1980 (a1, a2, a3, and a4), 1981–2000 (b1, b2, b3, and b4), 2001–2015 (c1, c2, c3, and c4) and 1961–2015 (d1, d2, d3, and d4) are compared. The coefficient of variation (i.e., the average value divided by the standard deviation) is used as the test statistic here. Most of the stations (blue dots) do not pass the Monte Carlo test at the *p* = 0.05 confidence level. The data on all of the available days are used.

Method 2 allows us to investigate the characteristics of the holiday effect at an intra-annual scale. The percentage of samples whose test statistic are larger than that of the 7-day holiday ΔT series, is regarded as the *p*-value. The FDR approach at *p* = 0.01 or *p* = 0.05 level is also applied to these *p*-values to obtain the significant results. When the test statistic used for the significance test is the coefficient of variation (i.e., the average value of Δ*T*_*mean*_, Δ*T*_*max*_, Δ*T*_*min*_ or the ΔDTR divided by its standard deviation), most of the stations (>88.3%; see supplementary Table S2) do not pass the Monte Carlo test at *p* = 0.01 or *p* = 0.05 level. The few stations that yield significant results are grouped regionally (see Fig. 4). When the test statistic is the range of ΔT (i.e., the difference between the maximum and minimum holiday effect values calculated using Δ*T*_*mean*_, Δ*T*_*max*_, Δ*T*_*min*_ or the ΔDTR), the holiday effect values obtained for all of the stations are insignificant. These differences in the significant results may be related to the different test statistics employed for the significance testing.

When the significance of the holiday effect is tested at the inter-annual scale using the *t*-tests, most stations (>99%; see supplementary Table S1) does not pass the significance test at the *p* = 0.05 confidence level. The Monte Carlo test on the holiday effects at an intra-annual scale confirms this result, with more stations failing to pass the significance test at the *p* = 0.05 confidence level. The use of different test statistics also permits us to evaluate the holiday effect in different ways.

### Determining Factors of the Holiday Effect

It has been shown that the ΔT values obtained during the week of the Chinese New Year holiday are not statistically significant at an intra-annual scale. The features of daily oscillations are analyzed for each station using the method with a combination of EEMD and the Hilbert transform (see Methods Section for details).

**Figure 5:**
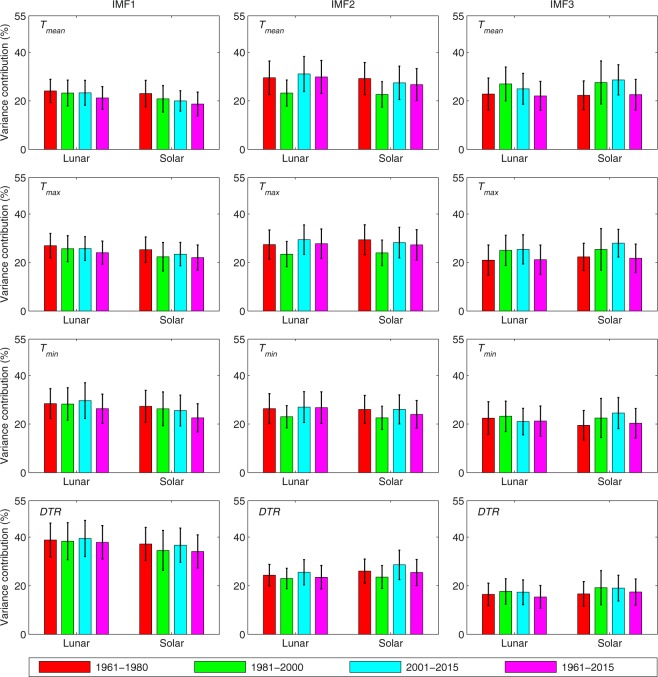
The average values (bars) and standard deviations (whiskers) of the variance contributions from the dominant IMFs that are decomposed from the ΔT data series of *T*_*mean*_, *T*_*max*_, *T*_*min*_ and the DTR. The ΔT data series are decomposed according to both the lunar calendar and the solar calendar, resulting in two types of data series. Different periods, i.e., 1961–1980, 1981–2000, 2001–2015 and 1961–2015, are compared, and the data on all of the available days are used.

Eight primary IMFs are obtained for each station after decomposing the daily series of Δ*T* (see Fig. S1). IMF1 through IMF7 contain information on the different signal sources of the Δ*T*_*min*_ value, including their frequencies. IMF8 denotes the trend in the data series. The contribution from the variance in each IMF is calculated for each station by dividing the variance in each IMF by the sum of all of the variances from all of the IMFs. Figure 5 shows the average values and standard deviations of the variance contributions in the dominant IMFs that are decomposed from the ΔT data series of *T*_*mean*_, *T*_*max*_, *T*_*min*_ and the DTR.

IMF1 through IMF3 are regarded as the dominant IMFs because they account for 72.9–82.2% of the oscillations in ΔT. During the period from 1961 to 2015, the variance contributions of each dominant IMF in Δ*T*_*mean*_, Δ*T*_*max*_ and Δ*T*_*min*_ range from 21.1% to 29.8% (see Fig. 5). The variance contribution of IMF1 in the ΔDTR during the period from 1961 to 2015 is 37.8%, whereas those of IMF2 and IMF3 are 23.5% and 15.4%, respectively. This result shows that weekly oscillation contributes more to ΔDTR than that of Δ*T*_*mean*_, Δ*T*_*max*_ and Δ*T*_*min*_, however, absolute values of holiday effect of the DTR may be substantially lower than that of Δ*T*_*mean*_, Δ*T*_*max*_ and Δ*T*_*min*_. The mechanism for this difference need further studies. Moreover, the variance contributions of the dominant IMFs during the three separate periods (1961–1980, 1981–2000 and 2001–2015) vary only slightly relative to those over the whole period (1961–2015). The standard deviations in the variance contributions among all of the stations vary from 4.2% to 7.6%.

**Figure 6:**
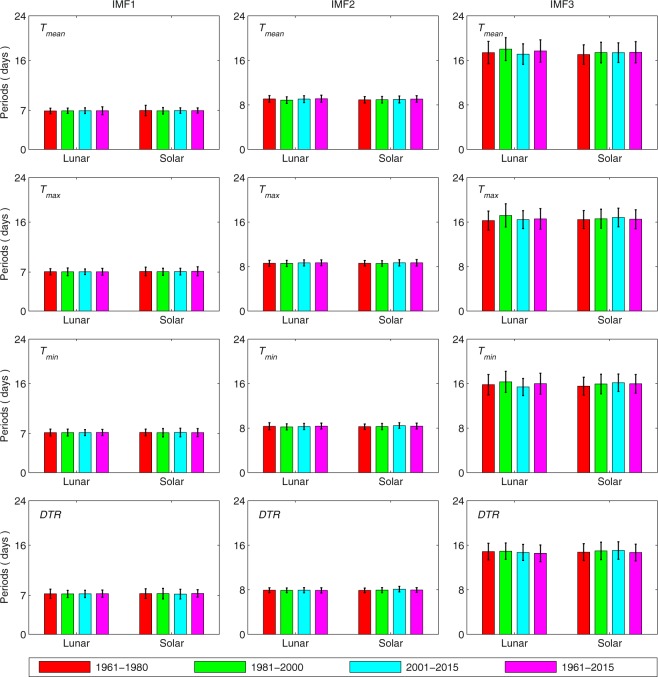
The periods of the dominant IMFs that are decomposed from the ΔT data series of *T*_*mean*_, *T*_*max*_, *T*_*min*_ and the DTR. The ΔT data series are decomposed according to both the lunar calendar and the solar calendar, resulting in two types of data series. Different periods, i.e., 1961–1980, 1981–2000, 2001–2015 and 1961–2015, are compared, and the data on all of the available days are used.

Figure 6 shows the period of each dominant IMF during the different time periods. During the period from 1961 to 2015, the period of IMF1 is 6.9–7.3 days within Δ*T*_*mean*_, Δ*T*_*max*_, Δ*T*_*min*_ and the ΔDTR according to the lunar calendar. For IMF2, the periods of Δ*T*_*mean*_, Δ*T*_*max*_, Δ*T*_*min*_ and the ΔDTR are 9.1 days, 8.7 days, 8.4 days and 7.9 days, respectively. The periods of both Δ*T*_*min*_ and the ΔDTR are approximately 8 days. IMF3 has a period of 17.7 days in Δ*T*_*mean*_ and periods of 16.6 days, 16.0 days and 14.5 days in Δ*T*_*max*_, Δ*T*_*min*_ and the ΔDTR, respectively. The standard deviations of the periods of IMF1 and IMF2 are 0.4–0.7 days, and the standard deviation is 1.5–2.0 days for the period of IMF3. The periodic features during the three separate periods (1961–1980, 1981–2000 and 2001–2015) are similar to those over the whole period (1961–2015).

**Figure 7:**
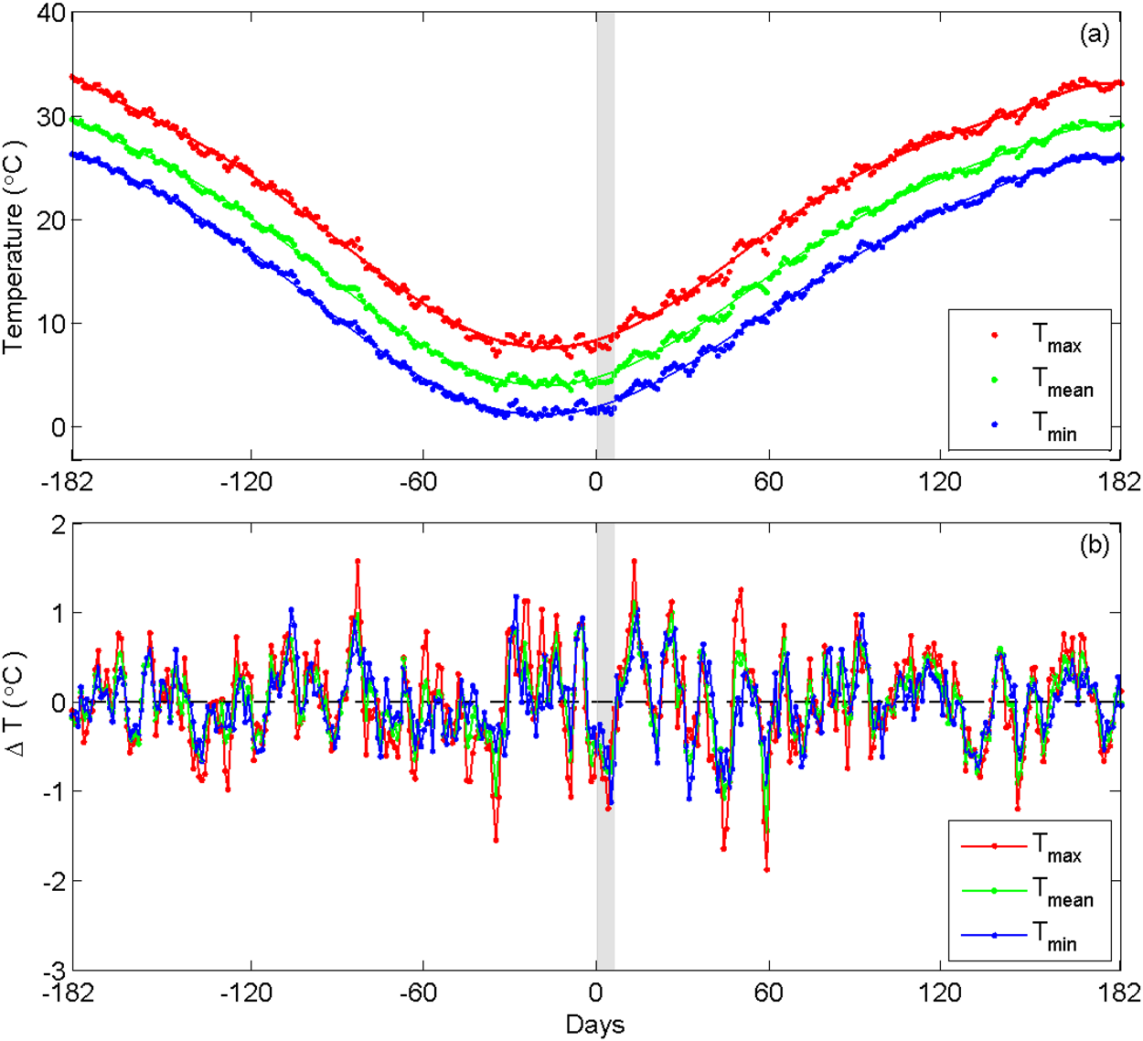
(**a**) The long-term (1961–2015) daily mean values and the values derived from a polynomial fit and (**b**) the daily deviations (ΔT) in *T*_*mean*_, *T*_*max*_ and *T*_*min*_ at station Anqing in Anhui Province. 9^th^ polynomial of order is used here. The slight gray shading covers the week of the Chinese New Year (days ‘0’ through ‘+6’), and the 60 days before and after the first day are referred to as days ‘−60’ and ‘+60’, respectively.

To evaluate the validities of the method used in Method 2 (see Fig. 7), the first day of the Chinese New Year is replaced with the solar New Year’s Day, which is accordingly defined as day ‘0’ in the polynomial regression. The abovementioned data processing scheme is repeated to investigate the differences that originate from sorting the data according to the lunar and solar calendars. Figures 5 and 6 compared the variance contributions and periods of the dominant IMFs according to the solar calendar, respectively. Figures 5 and 6 show similar results, indicating that sorting data methods do not influence the results significantly.

### The Holiday Effect on Dry Days

To further uncover the primary cause of the holiday effect on the air temperatures, the data processing scheme is repeated using only the data collected on dry days. The relevant figures are shown in Figs S2–S6. The average holiday effect and the associated significance tests of the results are shown in Tables 1, S1 and S2.

The variability in the holiday effect still exists within the different periods after excluding the data collected on precipitation days. During the period from 1961 to 1980, *T*_*mean*_, *T*_*max*_ and *T*_*min*_ all exhibit cooling holiday effects throughout most of the regions of China when only data on dry days are used. The corresponding simple spatial averages are approximately −0.41 °C to −0.49 °C (see Figs S2 and S3, Table 1) and are weaker than those obtained using the data on all of the available days. In the period of 1981–2000, the cooling holiday effects in *T*_*mean*_, *T*_*max*_ and *T*_*min*_ become uniformly stronger and there is still no cooling holiday effect observed in the DTR. In the period of 2001–2015, the cooling holiday effect in *T*_*mean*_ and *T*_*min*_ becomes weaker, whereas *T*_*max*_ and the DTR exhibit warming holiday effects, the variation is nearly equal to that using the data on all of the available days.

Spatial differences are also observed when the data excluding precipitation days are used. In the period of 1961–1980, warming holiday effects are observed in *T*_*max*_ and the DTR in the southeastern region. Moreover, the spatial patterns of the holiday effect are similar to those obtained when the data from all of the available days are used. Although a strong cooling holiday effect and a weak warming holiday effect are both observed on dry days, these effects fail to pass the statistical significance test at most stations (>79.3%, see Tables S1 and S2), which yield an equivalent result to the significance tests using the data on all of the available days.

## Discussion

The reduction in human activity during the Chinese New Year holiday leads to the reduction of waste heat and aerosol concentrations from traffic and industrial processes. The previous analyses found the cooling effect of Chinese New Year and other “golden week” holidays, and attributed it to population movement and the impact of the aerosols emission in eastern part of the country^32–34^. The reduction of waste heat causes both *T*_*max*_ and *T*_*min*_ to decrease; however, the reduced aerosol concentrations increase *T*_*max*_ and decrease *T*_*min*_^35^. The impacts of these changes on *T*_*max*_ partly cancel each other out, but their impacts on *T*_*min*_ do not. Therefore, if anthropogenic impact is the main cause of the holiday effect, the cooling holiday effect in *T*_*min*_ should increase over time due to the rapid increase in anthropogenic activity subsequent to 1978^31^. Moreover, the cooling holiday effect in *T*_*max*_ should be weaker than that in *T*_*min*_ due to the cancelling effects of changes in waste heat production and aerosol concentrations on *T*_*max*_.

However, the results obtained using the data on all of the available days show that the cooling holiday effect in *T*_*max*_ becomes weaker from the years 1961–1980 to the years 1981–2000. Moreover, *T*_*max*_ shows a warming holiday effect during the period from 2001 to 2015. The cooling holiday effect associated with *T*_*min*_ becomes stronger from the years 1961–1980 to the years 1981–2000 but decreases from the years 1981–2000 to the years 2001–2015. These variations in the holiday effect in both *T*_*max*_ and *T*_*min*_ lead to opposite holiday effects in the DTR, which shows a cooling holiday effect during the period from 1961 to 1980 and a warming holiday effect during the period from 2001 to 2015. This phenomenon is contrary to the pattern one might expect if only anthropogenic impacts alone were involved.

From the years 1961–1980 to the years 1981–2000, the national average cooling holiday effect in *T*_*mean*_ increases slightly and it migrates southeastward. Moreover, the cooling holiday effect in Xinjiang Province changes to a warming effect, which is a phenomenon that also occurs with regard to *T*_*max*_. Although this phenomenon might be related to the consistent advection of particulates from adjacent countries, this effect disappears in the period of 2001–2015.

The consistency in the holiday effects on *T*_*max*_ and *T*_*min*_ within the results obtained using only data on dry days indicates that anthropogenic impacts are not the primary cause of the holiday effect. Similar spatial patterns in the holiday effect are also observed after excluding precipitation days. However, a different pattern occurs within the southeastern region, where warming holiday effects are observed in *T*_*max*_ and in the DTR during the period from 1961 to 1980. This phenomenon is likely related to an increase in rainfall, which reduces *T*_*max*_ in the southeastern area.

The EEMD results reveal that annual time series of Δ*T*_*mean*_, Δ*T*_*max*_, Δ*T*_*min*_ and the ΔDTR at each station display oscillations with periods of approximately 7.1 days, 8.5 days and 16.2 days when all of the data are utilized (see Fig. 6). These oscillations account for approximately 75.6% of the variance in ΔT (see Fig. 5). When only dry days are considered, these periods are approximately 7.4 days, 7.8 days and 14.5 days (see Fig. S6), respectively, which account for approximately 80.4% of the variance in ΔT (see Fig. S5).

The period of each IMF of Δ*T*_*mean*_, *T*_*max*_ and Δ*T*_*min*_ is relatively spatially homogeneous. This homogeneity is likely related to large-scale meteorological variations, such as atmospheric oscillations. Studies have noted that oscillations with periods on the order of 7–9 days may be related to synoptic-scale Rossby waves, which have periods of approximately 7 days^36,37^. A period of 14.5~16.2 days can be regarded as a quasi-biweekly oscillation. Studies have identified key role of the Rossby waves and the NAO in the generation and maintenance of quasi-biweekly oscillations^20^.

An investigation of the correlations between the holiday effect and large-scale circulation patterns is therefore performed in this study. The values of the correlation coefficients describing the relationships between the time series of the NAO index and the inter-annual time series of the holiday effect values in *T*_*mean*_, *T*_*max*_ and *T*_*min*_ are 0.30–0.33 regardless of whether all of the available data or only the data on dry days are used. The negative and positive phase of NAO may be related to the changing signal of the holiday effect as measured by *T*_*mean*_, *T*_*max*_ and *T*_*min*_ with time periods.

Similarities between the holiday effect and atmospheric oscillations (i.e., Rossby waves and NAO) were detected, both of which demonstrate highly variable length and time scales. This result demonstrates that the so-called holiday effect is likely related to natural atmospheric oscillations. The spatial pattern of the holiday effect may be regarded as an extension of such oscillations. The central region and most of the southern regions of China are located in the troughs of these oscillations, whereas other areas, including the Tibetan Plateau, are located on the oscillation peaks^36,37^. The propagation of such oscillations leads to opposite reactions in regions in the peaks and troughs of the waves (Fig. S7)^19^.

Therefore, the so-called holiday effect is mainly attributed to the superposition of natural atmospheric oscillations with different periods (e.g., Rossby waves and the NAO). Although a positive correlation is found between the holiday effect and the NAO (which may be the reason for the changing signal of the holiday effect with time periods), current methods still cannot quantitatively distinguish the specific contributions from natural processes and human activities to the air temperatures during the holiday week. Further research is thus required.

## Methods

### Data and Study Area

Located on the western shore of the Pacific Ocean, China experiences a typical monsoon climate with cold, dry winters and hot, rainy summers. The terrain in China descends from the west toward the east, with elevations that reach up to 8336 m in the southwest (see Fig. 1). China is the world’s most populous country, and its economy and industry developed rapidly following the economic reform of 1978. Chinese Megacities have seen tremendous increases in energy consumption and air pollutant emissions^31^.

The Chinese New Year, which is the most important festival in China, has been celebrated for thousands of years. During the holiday season, almost all factories are shut down and people are off work. The official holiday typically lasts 7 days, and it begins on the first day of lunar January (the Chinese New Year’s Day) according to the Chinese lunar calendar. On the solar calendar (which refers to the Julian calendar in this study), this holiday falls in either January or February. To reconcile the differences between these two calendars, the Chinese lunar calendar has 12 lunar months in a typical year and 13 lunar months in a leap year. There are generally three leap years and two typical years in a five-year period.

In this study, the holiday effect of the Chinese New Year is evaluated using meteorological observations of *T*_*mean*_, *T*_*max*_, *T*_*min*_ and the DTR collected at 2200 stations in China from 1961 to 2015 (see Fig. 1). The daily *T*_*mean*_ represents the average of four regularly-timed observations collected at 02:00, 08:00, 14:00 and 20:00 Beijing local time. The datasets, which were obtained from the China Meteorological Administration, pass quality control standards.

Apart from the basic data quality control, the inhomogeneity of the datasets has also been considered^38–40^, which was mainly caused by the relocation of stations and instruments replacements^41,42^. In order to reduce the impact of this issue, the RHtest V4 was applied to check shifts in time series and adjust them by quantile-matching (QM) adjustments with reference series and metadata for each station^43,44^, The detailed process of this method was described in previous studies^45^. This method has also been demonstrated to efficiently adjust the shifts in time series caused by changes of location of stations, instruments and observation surroundings^46,47^. Moreover, only stations with observational records that are at least 82% complete are used in the individual data analysis. 82% is used as the threshold to obtain enough stations in each part of the data analysis and increase the reliability of the results. Approximate 2200 stations satisfy this requirement.

### Methods Used to Evaluate the Holiday Effect

In this study, two methods are used to evaluate the holiday effect on air temperatures during the week of the Chinese New Year holiday. Method 1 is a traditional method that directly compares the temporally-averaged values of *T*_*mean*_, *T*_*max*_, *T*_*min*_ and the DTR of the holiday week (a 7-day period) with those of the work week immediately preceding and following the holiday week (i.e., the 7 days preceding the holiday and the 7 days following the holiday) during each year, and it has been used in similar studies^32–34,48^. This method is used to allow direct comparison with the previous studies.

Method 2 is employed for a comparison with Method 1. In this method, the daily temperatures are first sorted according to the Chinese New Year’s Day (day ‘0’) for each station, as shown in Fig. 7, where the days from ‘0’ to ‘+6’ denote the holiday week and the days that fall 60 days before and after the Chinese New Year’s Day are denoted as ‘−60’ and ‘+60’, respectively. The multi-year averages of *T*_*mean*_, *T*_*max*_ and *T*_*min*_ are calculated for each day (dots in Fig. 7a) collapsed across all study years, these averages capture seasonal cycles of air temperatures throughout an entire year. Studies have shown that the variance in daily temperature will be overestimated if its seasonal cycle is ignored^49^. Therefore, functions that compute polynomial regressions (shown as curves in Fig. 7a) are used to remove the seasonal cycles of temperatures.

In Method 2, 9^th^ polynomial order is used to improve the regression results. The regression sufficiently capture the seasonal cycles, with a correlation coefficient of 0.98, and a standard error less than 0.3 °C. The daily deviations in air temperatures (ΔT, Fig. 7b) are obtained by removing their regressed seasonal cycle. This procedure is accomplished by subtracting the fitted temperature series (lines in Fig. 7a) from the original temperature series (dots in Fig. 7a). The average of the obtained ΔT during the holiday week (i.e., days ‘0’ through ‘6’) is regarded as the holiday effect of air temperature (see Fig. 7b). Apart from the holiday effect of *T*_*max*_, *T*_*min*_ and *T*_*mean*_, the holiday effect of the DTR is also calculated because several researchers have pointed out an anthropogenic impact on the DTR^50,51^. The holiday effect of the DTR is defined as the difference between the averaged DTR and the fitted DTR (the difference between the fitted *T*_*max*_ and the fitted *T*_*min*_ obtained from the polynomial regression) during the Chinese New Year Holiday.

### Statistical Tests of the Holiday Effect

Two different tests were used to assess the statistical significance of the holiday effect on air temperatures. Method 1 allows us to test the significance of the holiday effect at an inter-annual scale for each station using a single two-tailed *t*-test. A holiday effect value is obtained at each station for every year. The null hypothesis is that the inter-annual variations in the holiday effect at each station resemble a normal distribution with a mean of zero.

Method 2 allows us to investigate the multi-year mean holiday effect (collapsed across all study years) at an intra-annual scale (see Fig. 7b). The cooling effect during the holiday is not unique because oscillations are also observed in ΔT (see Fig. 7) in the context of a year. Therefore, the significance of the holiday effect over the whole year is doubtful. A Monte Carlo-type bootstrapping test^52–55^ is applied to the ΔT series for each station to examine the significance of the holiday effect (see Fig. 7b). The 7-day holiday period ranges from days ‘0’ to ‘+6’, and a random function is used to generate a ΔT series on 7 consecutive days from the remaining part of the original ΔT data (excluding the 7-day holiday weeks).

The test statistics obtained from the Monte-Carlo-produced 7-day ΔT series are then compared with those of the 7-day holiday ΔT series. This procedure is repeated 5,000 times for each station. The *p*-values are calculated as the proportion of test statistics that exceed those obtained during the 7-day holiday week during the 5,000 iterations. Following Earl *et al*.^54^, two types of test statistics are calculated, namely, the coefficient of variation (i.e., the average value of ΔT divided by its standard deviation) and the range of ΔT (i.e., the difference between the maximum and minimum values).

Studies have pointed out that strong spatial correlations exist in air temperature data, which may lead to an evaluation of field significance (the joint statistical significance) in multiple hypothesis tests^56^. In order to evaluate this issue, the false discovery rate (FDR) approach, which controls the expected proportion of null hypotheses that are mistakenly rejected (i.e., the proportion of rejected null hypotheses for which the null hypothesis is true), is applied to the *p*-values obtained from multiple hypothesis tests^57^. It has been demonstrated that the FDR is directly applicable to the field significance problem^53,56^, and has been applied to a variety of climate studies^58^. The FDR approach at *p* = 0.01 or *p* = 0.05 level is applied to the *p*-values to increase the chances that significant results in this study are not an evaluation of “field significance”.

### Decomposition Method for the Air Temperature Oscillations

The method used here combines ensemble empirical mode decomposition (EEMD) with a Hilbert transform to further analyze the air temperature oscillations. This method is highly effective for decomposition^59^ and is widely employed in geophysical research^60^. The EEMD is a refinement of the original empirical mode decomposition (EMD) method, which decomposes an original signal into a finite number of intrinsic mode functions (IMFs). Each IMF contains the local features of the original signal at different time scales, and the number of IMFs is positively correlated with the temporal scale of the data series^61^. The instantaneous frequency of each IMF can be obtained by performing a Hilbert transform, and the average period of each IMF can be easily calculated from the frequency.

After analyzing the holiday effect using data on all available days, the data processing was repeated using only data collected on dry days. In other words, the data collected during all precipitation days (i.e., those with daily cumulative precipitation values ≥ 0.1 mm, as measured from 8 p.m. to 8 p.m., local time) were excluded. Precipitation and clouds can decrease *T*_*max*_ and increase *T*_*min*_^62^, which may impact the inferred magnitude of the holiday effect on the air temperatures. Therefore, the holiday effect is also examined using only data from dry days.

## Electronic supplementary material

Supplementary Information
