# Supplementary material for: Exploring the Holiday Effect on Air Temperatures
Source: Sci Rep. 2018 Dec 18;8:17943. doi: 10.1038/s41598-018-36351-x (PMC6298989; doi:10.1038/s41598-018-36351-x)
Supplement: Supplementary file 1 — Supplementary Information [file 41598_2018_36351_MOESM1_ESM.docx]

*Scientific Reports*

Supporting Information for

Exploring the Holiday Effect on Air Temperatures

Shaojing Jiang^1, 2^ and Kaicun Wang^1*^

^1^State Key Laboratory of Earth Surface Processes and Resource Ecology, College of Global Change and Earth System Science, Beijing Normal University, Beijing, 100875, China

^2^School of Forestry and Environmental Studies, Yale University, New Haven, Connecticut, 06511, USA

**Contents of this file**

- Two tables S1-S2
- Seven figures S1-S7

Table S1. The percentage of significant stations at *p*=0.01 or *p*=0.05 level, as obtained using *t*-tests in the result of Method 1. The null hypothesis is that the temporal series of the holiday effect during each different period exhibited a normal distribution with a mean of zero. The results obtained using the data on all of the available days and on dry days only are compared.

| **Days** | **Temperature** | **1961-1980** | | **1981-2000** | | **2001-2015** | | **1961-2015** | |
| --- | --- | --- | --- | --- | --- | --- | --- | --- | --- |
|  |  | ***p*=0.01** | ***p*=0.05** | ***p*=0.01** | ***p*=0.05** | ***p*=0.01** | ***p*=0.05** | ***p*=0.01** | ***p*=0.05** |
| **All days** | ***T_mean_*** | 0.0% | 0.01% | 0.0% | 0.00% | 0.0% | 0.00% | 0.0% | 0.00% |
|  | ***T_max_*** | 0.0% | 0.00% | 0.0% | 0.00% | 0.0% | 0.00% | 0.0% | 0.04% |
|  | ***T_min_*** | 0.0% | 0.02% | 0.0% | 0.00% | 0.0% | 0.04% | 0.0% | 0.00% |
|  | ***DTR*** | 0.0% | 0.00% | 0.0% | 0.00% | 0.0% | 0.00% | 0.0% | 0.01% |
| **Dry days** | ***T_mean_*** | 0.0% | 0.00% | 0.0% | 0.00% | 0.0% | 0.03% | 0.0% | 0.00% |
|  | ***T_max_*** | 0.0% | 0.00% | 0.0% | 0.00% | 0.0% | 0.00% | 0.0% | 0.00% |
|  | ***T_min_*** | 0.0% | 0.01% | 0.0% | 0.00% | 0.0% | 0.00% | 0.0% | 0.00% |
|  | ***DTR*** | 0.0% | 0.00% | 0.0% | 0.00% | 0.0% | 0.00% | 0.0% | 0.00% |

Table S2. The percentage of significant stations at *p*=0.01 or *p*=0.05 level, as obtained using the Monte Carlo test in the result of Method 2 throughout the year. The coefficient of variation of △T and the range of △T are the two test statistics used to investigate the significance of the holiday effect. The results obtained using data on all of the available days and on dry days only are compared.

| **Days** | **Test statistic** | **Temperature** | **1961-1980** | | **1981-2000** | | **2001-2015** | | **1961-2015** | |
| --- | --- | --- | --- | --- | --- | --- | --- | --- | --- | --- |
|  |  |  | ***p*=0.01** | ***p*=0.05** | ***p*=0.01** | ***p*=0.05** | ***p*=0.01** | ***p*=0.05** | ***p*=0.01** | ***p*=0.05** |
| **All days** | **Coefficient of variation** | ***T_mean_*** | 9.6% | 10.4% | 2.5% | 2.5% | 10.2% | 11.9% | 5.2% | 6.2% |
|  |  | ***T_max_*** | 9.8% | 10.4% | 3.0% | 3.2% | 10.0% | 12.7% | 5.0% | 5.7% |
|  |  | ***T_min_*** | 9.7% | 11.4% | 3.9% | 4.0% | 10.7% | 12.7% | 7.3% | 8.3% |
|  |  | ***DTR*** | 8.8% | 9.7% | 8.8% | 9.5% | 10.5% | 13.0% | 6.9% | 8.4% |
|  | **Range** | ***T_mean_*** | 0.0% | 0.0% | 0.0% | 0.0% | 0.0% | 0.0% | 0.0% | 0.0% |
|  |  | ***T_max_*** | 0.0% | 0.0% | 0.0% | 0.0% | 0.0% | 0.0% | 0.0% | 0.0% |
|  |  | ***T_min_*** | 0.0% | 0.0% | 0.0% | 0.0% | 0.0% | 0.0% | 0.0% | 0.0% |
|  |  | ***DTR*** | 0.0% | 0.0% | 0.0% | 0.0% | 0.0% | 0.0% | 0.0% | 0.0% |
| **Dry days** | **Coefficient of variation** | ***T_mean_*** | 19.2% | 19.5% | 11.8% | 11.8% | 13.6% | 13.8% | 12.0% | 12.7% |
|  |  | ***T_max_*** | 18.7% | 18.8% | 12.2% | 12.3% | 14.0% | 14.3% | 11.9% | 12.8% |
|  |  | ***T_min_*** | 20.3% | 20.7% | 13.4% | 13.4% | 15.6% | 15.7% | 13.1% | 13.6% |
|  |  | ***DTR*** | 19.2% | 19.7% | 16.9% | 17.4% | 15.7% | 15.8% | 14.2% | 15.1% |
|  | **Range** | ***T_mean_*** | 0.0% | 0.0% | 0.0% | 0.0% | 0.0% | 0.0% | 0.0% | 0.0% |
|  |  | ***T_max_*** | 0.2% | 0.2% | 0.3% | 0.3% | 0.1% | 0.1% | 0.0% | 0.0% |
|  |  | ***T_min_*** | 0.2% | 0.2% | 0.0% | 0.0% | 0.0% | 0.0% | 0.0% | 0.0% |
|  |  | ***DTR*** | 0.3% | 0.3% | 0.0% | 0.0% | 0.0% | 0.0% | 0.0% | 0.0% |

Fig. S1. The decomposition results for the △T values derived from *T_min_* at station Anqing in Anhui Province using ensemble empirical mode decomposition (EEMD) during 1961-2015. Eight intrinsic mode functions (IMFs) are obtained. IMF1 through IMF7 denote the dominant frequencies of the original signal and contain the information from different signal sources within the △T, whereas IMF8 denotes the trend in the data series.


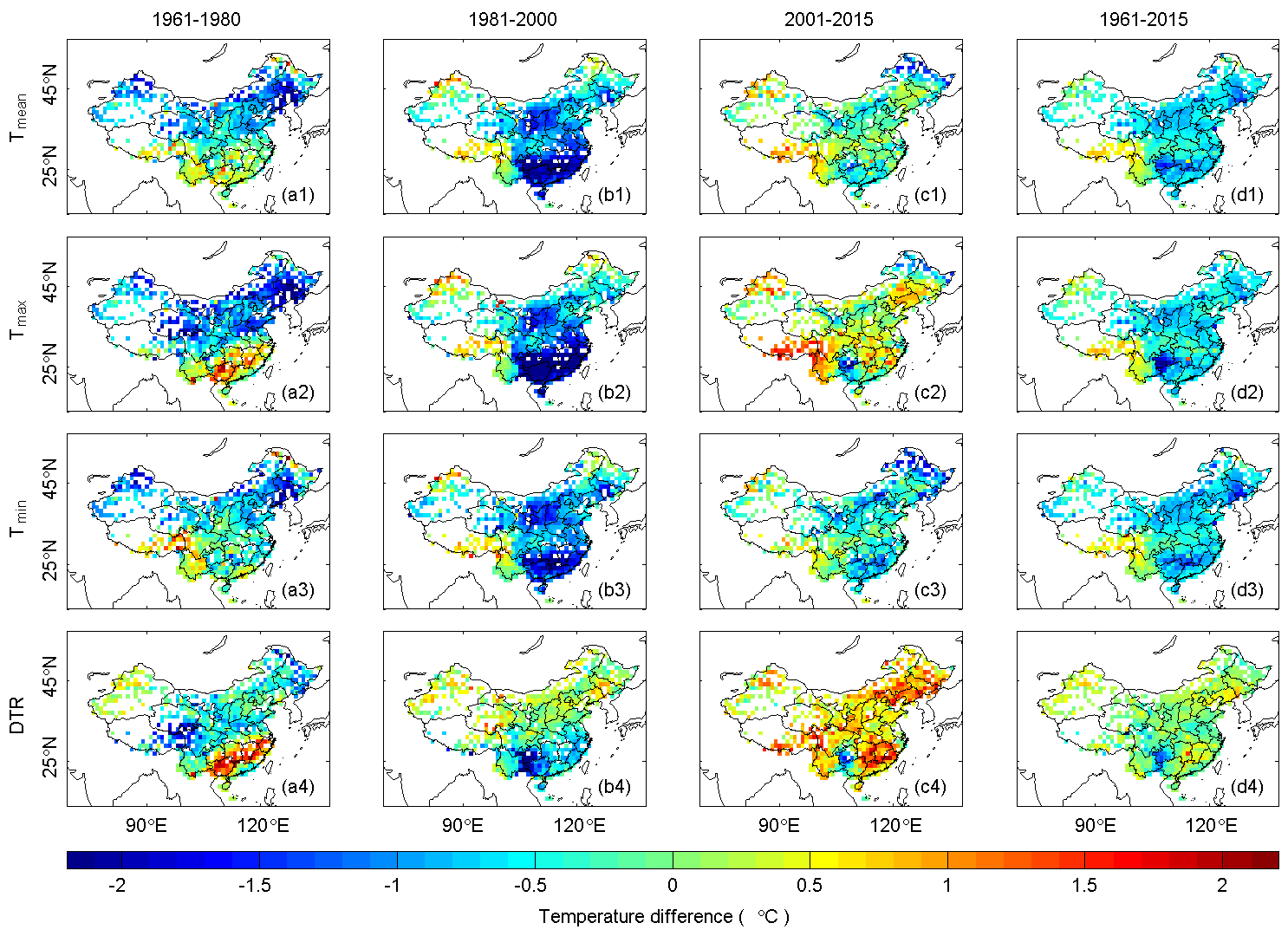


Fig. S2. Same as Fig. 2, except that only data on dry days are used instead of using the data on all of the available days.


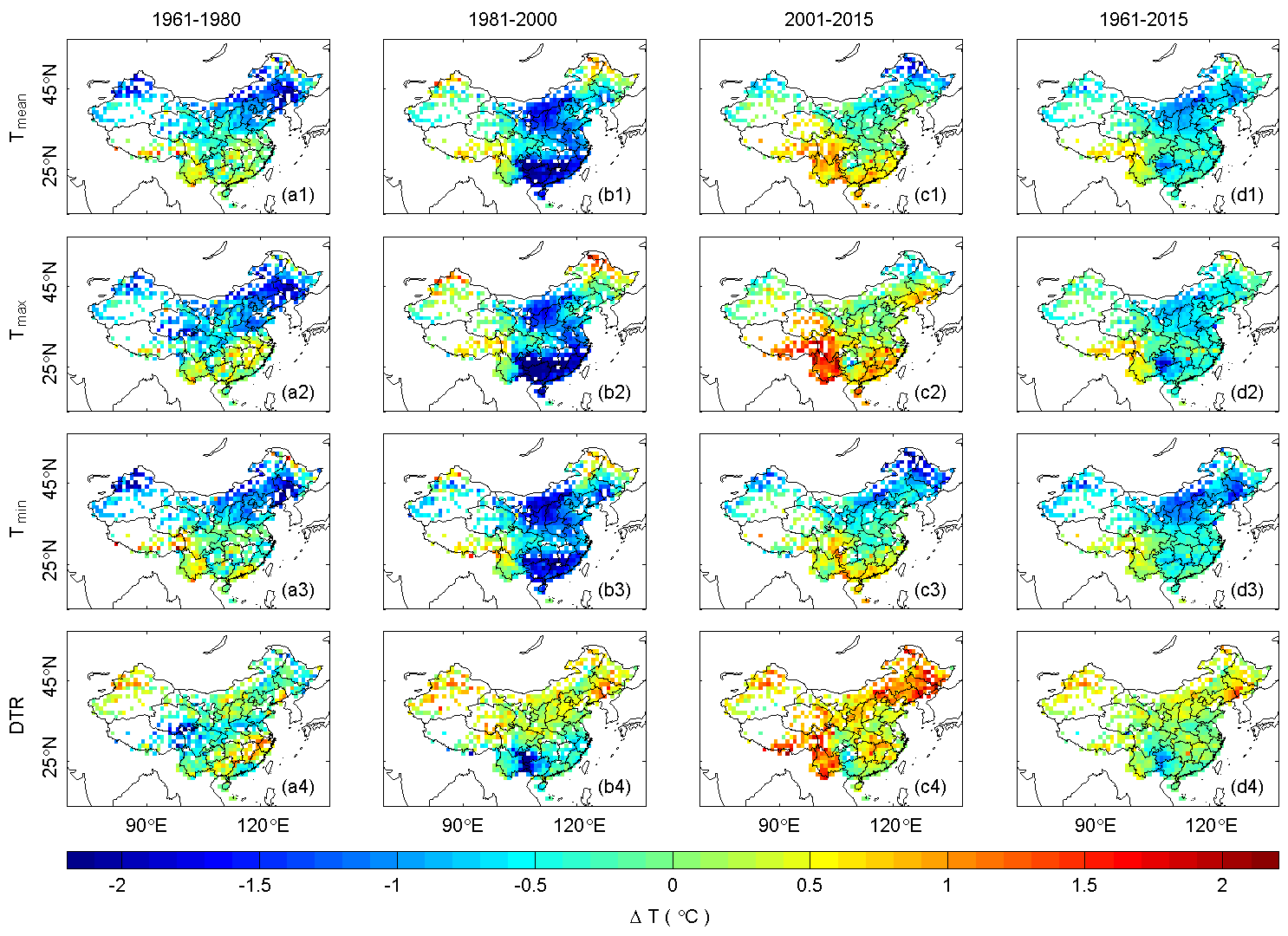


Fig. S3. Same as Fig. 3, except that only data on dry days are used instead of using the data on all of the available days.

Fig. S4. Same as Fig. 4, except that only data on dry days are used instead of using the data on all of the available days.

Fig. S5. Same as Fig. 5, except that only data on dry days are used instead of using the data on all of the available days.

Fig. S6. Same as Fig. 6, except that only data on dry days are used instead of using the data on all of the available days.


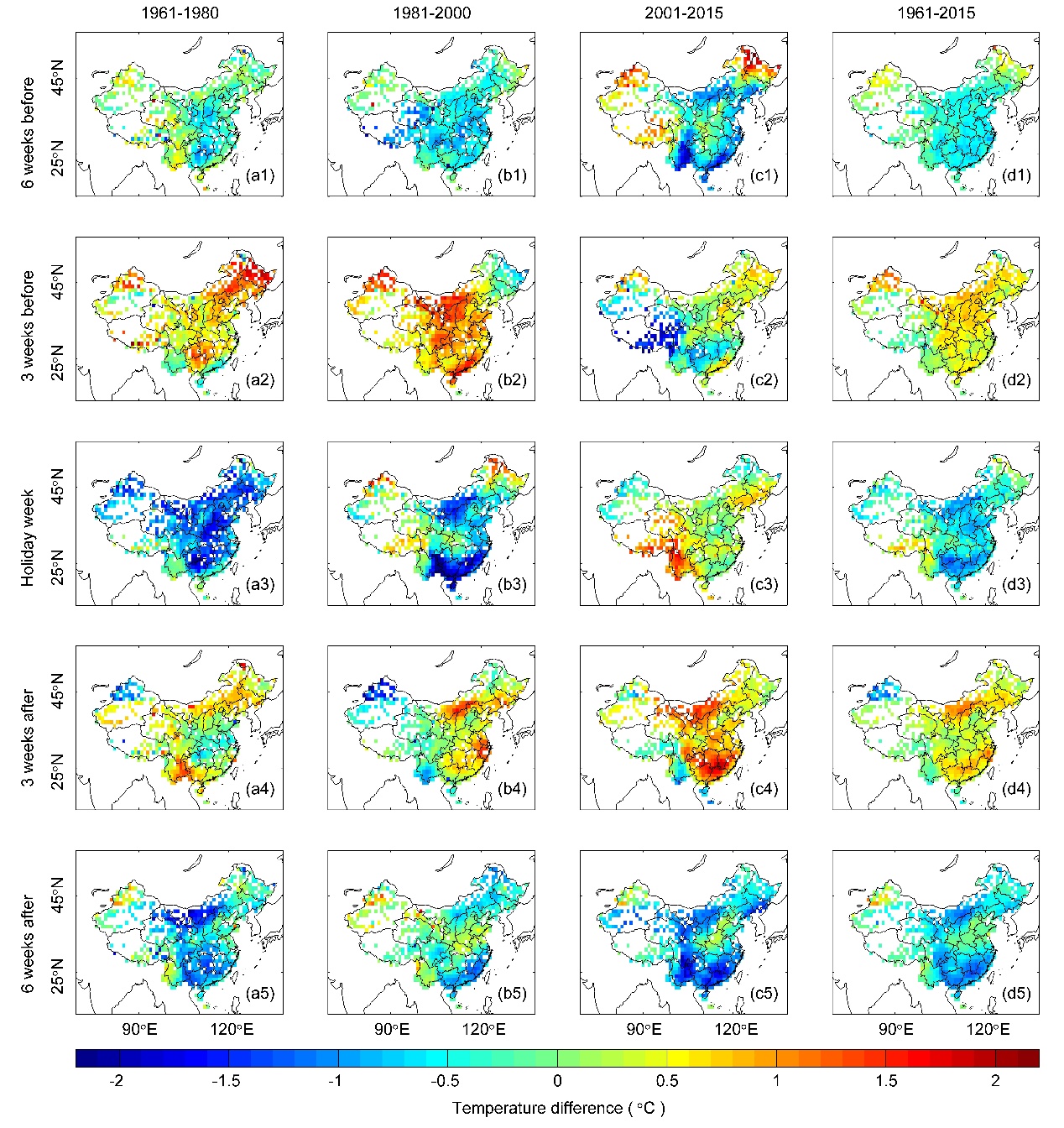


Fig. S7. Spatial distribution of averaged daily deviations of *T_max_* at different weeks in China using Method 2 during 1961-1980 (a1, a2, a3, a4, and a5), 1981-2000 (b1, b2, b3, b4, and b5), 2001-2015 (c1, c2, c3, c4, and c5) and 1961-2015 (d1, d2, d3, d4, and d5). The data on all of the available days are used.
